# Residual Kidney Function and Cause-Specific Mortality

**DOI:** 10.1016/j.ekir.2023.08.024

**Published:** 2023-08-24

**Authors:** John T. Daugirdas

**Affiliations:** 1 Division of Nephrology, Department of Medicine, University of Illinois College of Medicine, Chicago, Illinois, USA

See Clinical Research on Page 1989

It is well known that some degree of preserved residual kidney function (RKF) in patients with end-stage kidney disease is associated with higher rates of survival.^1^^,^^2^ It has also been found that loss of RKF is associated with a high mortality rate.^3^ There are several questions in this area that remain unanswered: (i) Is the reduced mortality with RKF found with all-cause deaths or is the association limited to deaths due to cardiovascular disease or infection? Is the presence of RKF associated with reduction in sudden cardiac deaths? (ii) What is the “dose-response” relationship between the level of RKF and survival benefit? (iii) What are the possible mechanisms for the higher survival rate when RKF is present? Is there lowering of cardiovascular stress because of the lower ultrafiltration rates typically found in patients with RKF? Do patients with RKF have a lesser degree of fluid overload than those with anuria? Is the survival benefit of RKF linked to less exposure to very high levels of serum potassium or phosphate, each of which has been associated with mortality, and in the case of potassium, with sudden death? (iv) Does presence of RKF allow for a more liberal diet, leading to improved nutrition and a lower prevalence of protein-energy wasting? (v) Does increased removal by RKF of high molecular weight and protein-bound uremic toxins meaningfully lower serum levels and thereby reduce inflammation and mitigate other pathology involved in the uremic syndrome?

No single study can answer all these questions, but the observational study by Okazaki *et al.*^4^ published in this issue of *Kidney International Reports* provides important and useful new insights. These investigators were able to identify 39,000 incident hemodialysis patients in whom residual kidney clearance of urea and urine volume had been measured approximately 60 days after starting hemodialysis. In one-third, repeat measurements were available, allowing for assessment of associations between the extent of decline in RKF or urine volume (over a 6-month period after enrollment) and mortality.

Okazaki *et al.*^4^ confirmed multiple previous reports, revealing that death rate was lower in patients who had retained some residual kidney clearance of urea at baseline. This survival association was found with noncardiovascular deaths, sudden cardiac death (SCD), and non-SCD cardiovascular deaths. With respect to a “dose-response” relationship for these associations, for noncardiovascular deaths, the data suggested a monotonically higher death rate in subgroups with renal clearance of urea below 6 ml/min per 1.73 m^2^, with a more pronounced higher death rate when this value was below 1.5. A plethora of adjustments, including case-mix, ultrafiltration rate, and a packet of laboratory values had little effect on the observed “dose-response” association. For SCD and non-SCD cardiovascular deaths, the dose-response relationship between renal urea clearance at baseline and mortality risk depended strongly on whether an adjustment for “laboratory values” was made. These included the normalized protein catabolic rate; predialysis blood hemoglobin; predialysis serum levels of albumin, creatinine, phosphate, iron saturation, bicarbonate, alkaline phosphatase, and ferritin; and the highest predialysis serum potassium level measured during the initial 3-month observation period. Body mass index was included in the “laboratory values” adjustment packet. If the “laboratory values” adjustments were excluded, the dose-response relationship between renal clearance of urea and cardiovascular deaths seemed to differ slightly from that observed with noncardiovascular deaths. Compared with patients with renal urea clearance >6.0 ml/min per 1.73 m^2^, the noncardiovascular death rate was already higher when renal urea clearance was less than 6.0, whereas for nonsudden cardiovascular deaths (non-SCD), the risk of death was higher only in the subgroups with renal urea clearance below 1.5 ml/min per 1.73 m^2^. For SCD, the death risk was higher in subgroups with renal urea clearances below 3.0 (including those in the 1.5–3.0 ml/min range).

The authors then did a similar “dose-response” analysis for the 3 categories of cause-specific mortality versus daily urine volume. Here, mortality risk seemed to be higher in subgroups in whom daily urine volume was below 900 ml/d. Results were similar for noncardiovascular death, SCD, and non-SCD cardiovascular death.

When evaluating the effect of a change in urine volume over the 6-month period after enrollment, an increase in urine volume was associated with a reduced death risk for all 3 mortality categories, whereas a reduction in urine volume was associated with a higher death risk for noncardiovascular death and for SCD. For non-SCD cardiovascular death, a reduction in urine volume was not clearly associated with a higher risk of death.

When trying to analyze mechanistic variables, including ultrafiltration rate, nutrition, potassium, and phosphate, the study made several interesting observations. In their Supplementary data Table S4, ultrafiltration rate, normalized protein catabolic rate, and serum potassium and phosphate are compared in subgroups based on different levels of daily urine output at baseline. The renal clearances of urea tracked daily urine output, as expected. One of the potential “benefits” of a high daily urine volume is a lower ultrafiltration rate during dialysis. When a given volume of fluid is ingested or generated from food during the week, fluid excreted by the kidneys no longer has to be removed during dialysis and the ultrafiltration rate is proportionately lowered. In this Supplementary Table S4, we find that in the subgroup with urine output <300 ml/d, ultrafiltration rate averaged 8.0 ml/h per kg, whereas in the subgroup with urine output >1200 ml/d, the average ultrafiltration rate was only 6.5. Similar differences were found among the urine volume subgroups for values of weekday and weekend interdialytic weight gains. With regard to nutrition/inflammation, serum albumin was slightly higher in the subgroups with higher daily urine output. The normalized protein catabolic rate was substantially higher in the subgroups with higher urine volume, averaging 0.80 g/kg/d when urine volume was <300 ml/d versus 1.11 when urine volume was >1200 ml/d, with relatively monotonically increased catabolic rate value for the urine volume subgroups in between.

One might have expected the predialysis serum potassium and phosphate values to be lower in the subgroups with higher urine volume, but they were not. Furthermore, the incidences of high predialysis serum potassium levels (>6.0 or >6.5 mmol/l) during the 3-month baseline period were similar in the different urine volume subgroups. The most logical explanation for this is that patients in the subgroups with higher urine volume were eating more and the increased food intake overrode any increased removal of potassium and phosphate due to RKF. So, were the benefits of increased renal clearance of urea at baseline on subsequent survival mediated by ultrafiltration, nutrition, potassium, or phosphate? Adjusting the mortality analyses for baseline ultrafiltration rate or incidence of high baseline serum potassium levels did not meaningfully change the associations between baseline renal urea clearance and the 3 categories of mortality.

In summary, the results of the study by Okazaki *et al.*^4^ are primarily confirmatory and suggest a broad-based benefit of increased RKF on survival. The association was not limited to a reduced rate of SCDs or of non-SCD cardiovascular deaths, but it was as or more prominent with noncardiovascular mortality. Cause-specific mortality due to infection was not analyzed and this would have been of interest. The exact mechanism whereby RKF is associated with a lower death risk remains incompletely defined, but it is most likely related to reduced serum levels of potential uremic toxins rather than to lower ultrafiltration rates or to lower incidence rates of severe hyperkalemia. Even small amounts of RKF can substantially lower serum levels of higher molecular weight toxins such as beta-2-microglobulin^5^ and protein-bound uremic toxins, which are not well removed by hemodialysis. Future exploration of the role of RKF and urine volume on hard outcomes should ideally include measurement of serum levels of such uremic toxins.

## Disclosure

The author declared no competing interests.
